# PROFESSIONAL STAFFING AND ROLES IN CARE TEAMS SERVING FRAIL ELDERS LIVING IN THE COMMUNITY: 22 SITE VISITS

**DOI:** 10.1093/geroni/igz038.257

**Published:** 2019-11-08

**Authors:** Barbara Roberge, Carie Michael, David I Auerbach, Peter Maramaldi, Karen Donelan

**Affiliations:** 1 Massachusetts General Hospital, Boston, Massachusetts, United States; 2 The Mongan Institute, Health Policy Research Center, Boston, Massachusetts, United States; 3 Massachusetts Health Policy Commission, Boston, Massachusetts, United States; 4 Simmons College School of Social Work, Boston, Massachusetts, United States

## Abstract

In 2017, as part of a study to understand the evolving roles of nurses, physicians and social workers in leading and working in teams, our interprofessional team explored 22 sites of care for frail elderly adults in five US regions (Chicago IL, Denver CO, Tampa/Orlando FL, San Diego CA, New England).. The purpose of these site visits was to understand the current range of models of care for frail elders living in community, the roles of health professionals within those care models, and to inform national measure development. We selected regions based on elder population density, scope of NP practice, and screened over 100 sites to identify physician, nurse and social work led teams. We included general primary care, PACE, academic geriatrics, home based primary care, assisted living, FQCHC, palliative care, mobile health. We interviewed 108 key informants. We found considerable variation in staffing/elders within each site type.

